# A pentacyclic triterpene natural product, ursolic acid and its prodrug US597 inhibit targets within cell adhesion pathway and prevent cancer metastasis

**DOI:** 10.18632/oncotarget.3261

**Published:** 2015-03-19

**Authors:** Liping Xiang, Ting Chi, Qiao Tang, Xiang Yang, Minrui Ou, Xiufen Chen, Xiaobo Yu, Jianzhong Chen, Rodney J.Y. Ho, Jingwei Shao, Lee Jia

**Affiliations:** ^1^ Cancer Metastasis Alert and Prevention Center, and Pharmaceutical Photocatalysis of State Key Laboratory of Photocatalysis on Energy and Environment, College of Chemistry, Fuzhou University, Fuzhou 350002, China; ^2^ School of Pharmacy, Fujian University of Traditional Chinese Medicine, Fuzhou, Fujian 350108, China; ^3^ Department of Pharmaceutics, University of Washington, Seattle, WA 98105, USA

**Keywords:** Anti-metastasis, ursolic acid, cell adhesion molecules, integrin, focal adhesion

## Abstract

Here we showed that ursolic acid (UA), a pentacyclic triterpene natural product, and its novel prodrug derivative US597 suppressed cancer cells adhesion, invasion and migration. This effect was accompanied by inhibition of focal adhesion signaling pathway including alterations in ICAM-1, VCAM-1, E-selectin, P-selectin, integrin α6β1, FAK, Src, paxillin and PTEN. While oral administration of UA or US597 increases survival rate of melanoma lung metastasis in C57BL/6 mice, US597 treatment extend the survival rate above that of UA. Immunohistochemical analysis revealed that US597 treatment regulates ICAM-1, a biomarker of metastasis. We did not detect side effects with US597 in mice such as weight loss, viscera tissues toxicity and blood cell abnormalities. Thus, UA and US597 are potential drug candidates for preventing cancer metastasis. Molecular and cellular study data suggest that UA and US597 modulate expression of cell adhesion molecules within focal adhesion signaling pathway leading to cancer cell motility.

## INTRODUCTION

Metastasis is one of the major causes of mortality in cancer patients, although surgical removal of tumors can enhance and prolong survival [1]. Cancer metastasis involves an intricate multiprocess cascade including cell adhesion, migration, invasion, cell-to-cell and cell-to-ECM (extracellular matrix) interactions. The major cause of cancer-related deaths is often due to tumor cells adhesion, invasion and migration, which results in the formation of distant metastasis.

The adhesion between tumor cells and vascular endothelial cells is one of the crucial procedures of cancer early metastasis [2]. The cell adhesion molecules (CAMs), such as vascular cell adhesion molecule-1 (VCAM-1), intercellular adhesion molecule-1 (ICAM-1) and endothelial cell selectin (E-selectin), which expressed by endothelial cells may play a critical role in the cancer metastasis [3]. There is strong evidence that blocking the expression and activity of CAMs can reduce the cancer metastasis [4–6], which indicated that anti-adhesion is an effective strategy for cancer metastasis inhibition. The integrin family of adhesion molecules, a specific class of adhesion receptors, is involved in various processes associated with adhesion, migration, invasion of tumor cells [7, 8]. Integrins and their downstream signaling partners (such as FAK, Src, paxillin and PTEN) play an important role in cell adhesion [9], and inhibition of integrins expression may have a beneficial effect on anti-metastasis therapy [10–13]. Among them, it has been shown that the α6β1 integrins plays a key role in hepatocellular carcinoma metastasis [11, 12]. Consequently, the therapy of anti-adhesion may be a direction that prevents the recurrence and metastasis of tumor, and it is necessary to develop novel effective drugs to inhibit cancer metastasis.

Ursolic acid (UA), 3β-hydroxy-urs-12-en-28-oic acid, is an ursane-type pentacyclic triterpenic acid found in a variety of plant, including medicinal herbs and edible plants especially in the leaves and berries of natural medicinal plants, such as bearberry, cranberry, rosemarry and in the protective wax-like coatings of apples, pears, prunes and other fruits. It exhibits comprehensive biologic properties in a variety of human diseases, including anti-inflammatory [14], anti-angiogenesis [15], anti-cancer [16, 17], anti-HIV [18] and antimalarial [19] activities. Among them, antiproliferative, proapoptotic, suppressed tumor invasion and anti-metastatic potential of ursolic acid have been reported in both *in vitro* cell culture systems and *in vivo* experiments [20, 21]. In a previous study, we showed that a novel UA derivative US597 has significant anti-tumor activities including anti-proliferation, induction of apoptosis, cell cycle arrest, mitochondrial inhibition and apoptosis/necrosis induction [22]. Because UA and most of its derivatives (UAs) are relatively non-toxic to normal cells [23], an important implication of these findings is that they might play a useful role in the treatment of cancer metastasis. However, little is known regarding the anti-adhesion and anti-invasion effects of UAs as well as their exact molecular mechanisms of actions and related pathways on tumor metastasis.

In the current study, we investigated the anti-metastasis effect of UA and its derivative US597 on the cell growth, adhesion, invasion and migration of SW620, B16-F10 and HepG2 cells *in vitro*, and further explored the underlying mocelular mechanism of UA/US597 on the IL-1β-induced cell-surface CAMs expression in HUVECs and integrin-mediated focal adhesion signaling pathway in tumor cells. Additionally, we evaluated whether UA/US597 had a potential for the prevention or treatment of cancer metastasis *in vivo* by the B16-F10/C57BL/6 mouse melanoma lung metastasis model.

## RESULTS

### Effect of UA/US597 on cell viability

**Figure 1:**
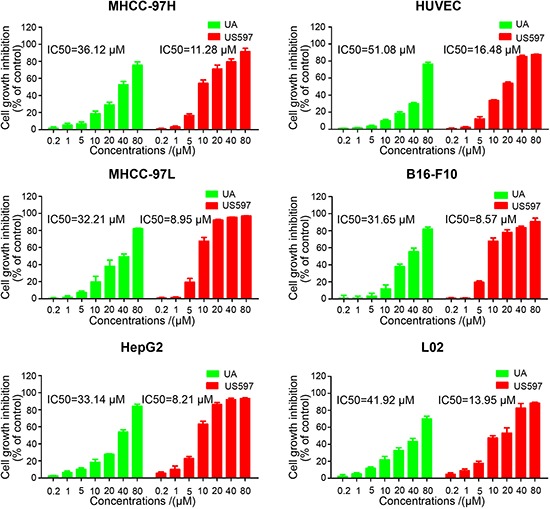
Inhibitory effect of UA/US597 on the proliferation of human hepatic cancer HepG2, MHCC-97H/L cells; melanoma B16-F10 cells, the normal human liver cell line L02 and HUVCEC cells The results shown were the mean of 3 parallel experiments for each concentration point.

To explore the metastatic chemopreventive function of UA/US597, we first examined cytotoxic effect against nine different cancer cell lines including MHCC-97H, MHCC-97L, HepG2, M619, MDA-MB-231, MCF-7, HT29, SW620 and B16-F10 after treatment with various concentrations of UA/US597 for 24 h, and the viability of cells was determined with MTT assays. As shown in Figure 1 and Supplementary Figure S1, the IC50 values for UA to suppress cell proliferation varied from 31.65–60.11 μM in nine cancer cell lines, and we found that US597 significantly inhibited cell proliferation in all 9 cell lines in a dose-dependent manner, the IC50 varied from 8.21 to 17.28 μM; HepG2 and B16-F10 cells were found to be more sensitive than other cancer cells as indicated by their IC50 value (HepG2, 8.21 μM; B16-F10, 8.57 μM).

To determine the cytotoxicity of UA and US597 on normal human cells, we conducted MTT assay in L02 and HUVEC cells after administration with indicated concentrations of compounds. UA and US597 sufficiently inhibited L02 cells only at concentrations of 41.92 and 13.95 μM, respectively. In the mean time, UA and US597 inhibited HUVEC cells viability at a much higher concentration with an IC50 value of 51.08 and 16.48 μM, respectively. By comparison, the cytotoxicity of UA or US597 was very low at the concentration of 0.2–5 μM. Based on the comparison, SW620, B16-F10 and HepG2 cells were then chosen for further studies to explore UA/US597 anti-metastasis *in vitro*.

### Effect of UA/US597 on the adhesion, invasion and migration of tumor cells *in vitro*

**Figure 2:**
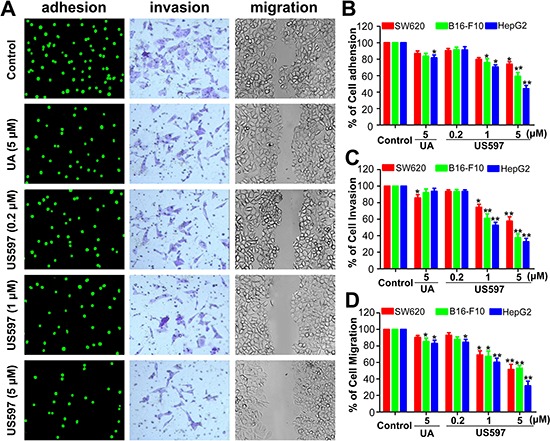
(A) The number of adherent HepG2 cells was photographed under the fluorescence microscope at × 200 magnification (left); b, phase micrograph of invading HepG2 cells were treated with UA or US597 (middle); c, phase micrographs of HepG2 cells were treated with UA or US597 at 24 h after monolayer wounding (right) **(B)** Quantitative analysis of the inhibition by UA/US597 on the adhesion of SW620, B16-F10 and HepG2 to HUVECs. **(C)** Cells invaded through the membrane were quantified. **(D)** Migrated cells were quantified by manual counting. Data are obtained from 3 separate experiments and bars represent the mean ± SD. * indicates *P* < 0.05 and ** means *P* < 0.01.

Tumor cells adhesion to the ECM is a fundamental step in cancer metastasis, we first evaluated the adhesion effect of UA/US597 on SW620, B16-F10 and HepG2 cancer cells to HUVECs at non-cytotoxic concentrations. The tumor cells were labeled with a fluorescent dye rhodamine 123, fluorescence microscope observation revealed that US597 interfered adhesion of HepG2 cells to HUVECs in a concentration-dependent manner (Figure 2A). To quantitatively examine the intervention by UA/US597 of tumor cells adhered to HUVECs, 10 fields of each well were randomly selected, and the adhered spots were counted. Compared with the control group, the adhesion rate of HepG2 cells was 84.83 ± 3.35% (*P* < 0.05) in UA-treated group, and the adhesion rate of SW620, B16-F10 and HepG2 cells was 73.93 ± 3.58%, 59.26 ± 2.29%, 44. 16 ± 4.22%, respectively, corresponding to 5 μM of US597 (Figure 2B), indicating that US597 may fit into a new class of therapy for the reduction of risk factors for cancer metastasis.

To determine whether UA/US597 affects the invasion and migration of SW620, B16-F10 and HepG2 cells, the invasion assay and the wound-healing assay were performed. In the transwell assay, UA/US597 decreased invaded cell number 24 h after drug treatment. The average number of invaded HepG2 cells in the control group was 88 ± 5, in UA group, the average number of invaded cells was 75 ± 3, and the number were 78 ± 3, 60 ± 5, and 28 ± 6 in US597 groups, respectively (Figure 2A). On the other hand, UA/US597 exhibition on invasion of the SW620 and B16-F10 cell lines through the transwell membrane at low concentrations suggesting its specific inhibition on cell invasion (Figure 2C).

In wound healing assay (Figure 2A and 2D), the speed of wound healing of HepG2 cells movement was significantly lower than that of control cells. The wound of HepG2 cells was almost closed 24 h after wounding, while the cells treated with various concentrations of US597 stayed wide apart, showing US597 inhibited cell motility dose-dependently. Whereas UA treated group showed less migration in wound area under the same condition. In addition, the inhibition on cell migration might be more effective on HepG2 cells compared to SW620 or B16-F10 cells. The wound healing assay demonstrated that the migration of HepG2 cells incubated with UA/US597 was significantly decreased compared with the control.

### Down-regulation the mRNA and protein expression of cell adhesion molecule by UA/US597 in HUVECs

**Figure 3:**
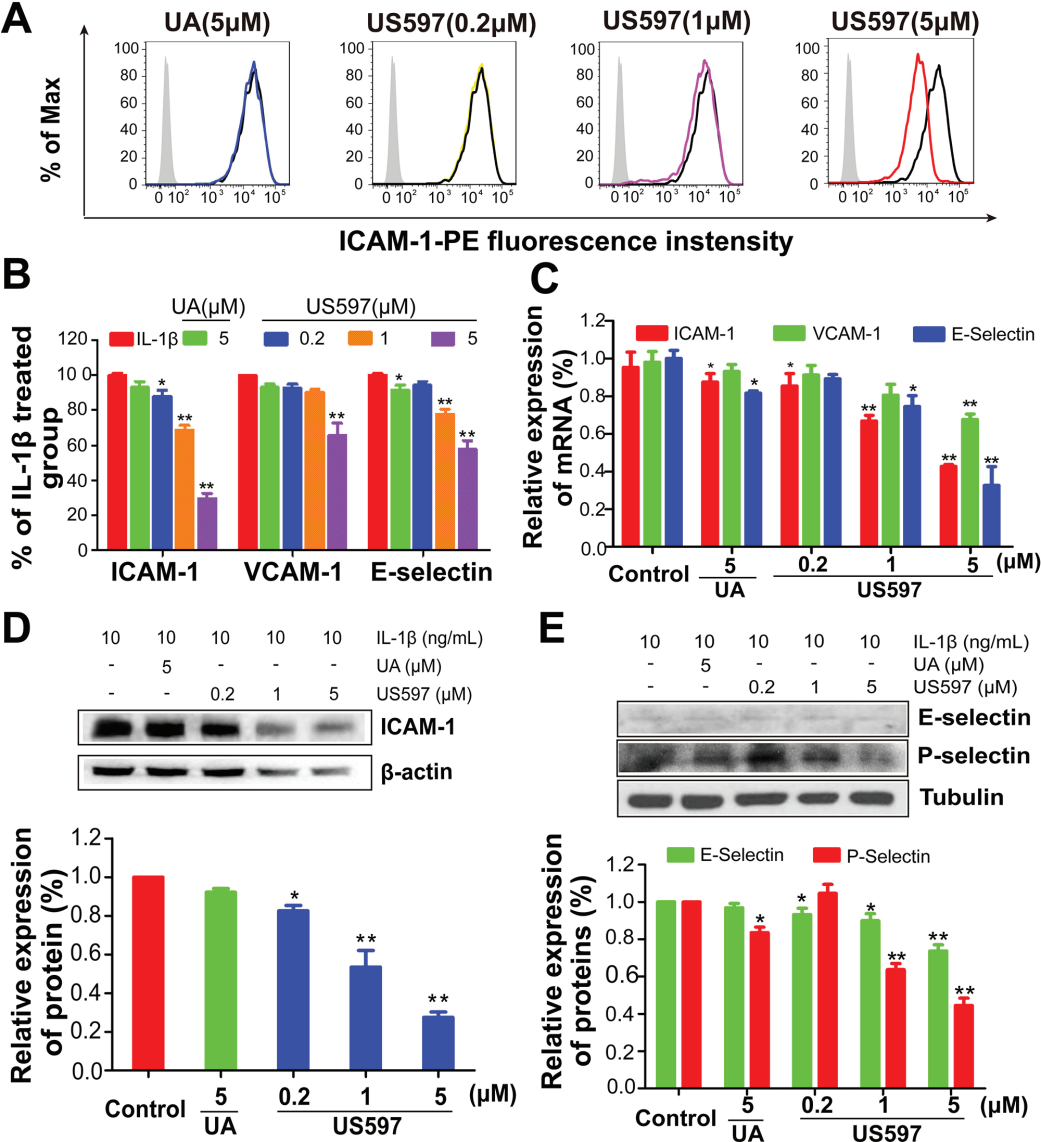
(A) Expression of ICAM-1 on HUVECs determined by flow cytometry, and (B) the inhibitory effect of UA/US597 on the expression of ICAM-1, VCAM-1 and E-selectin, silver area is an isotype control, blank curve as a control, blue curve represents UA (5 μM) treated group, yellow, purple and red line represents US597 treated group at concentrations of 0.2, 1 and 5 μM, respectively **(C)** Quantitative real-time PCR assay of the inhibitory effect of UA/US597 on the mRNA expression of ICAM-1, VCAM-1 and E-selectin. The expression of ICAM-1 **(D)**, E-selectin and P-selectin **(E)** on HUVECs were analyzed by Western blotting. Data were expressed as the percentage of control. Bars represent the mean ± SD (*n* = 3); *P < 0.05 and **P < 0.01.

To verify the accurate molecular targets that UA/US597 affects the adhesion, invasion and migration of HepG2 cells, the effects of UA/US597 on the mRNA and protein expression by HUVECs of ICAM-1, VCAM-1 and E-selectin were evaluated by qRT-PCR, flow cytometry and western blot. The HUVECs were pretreated with 1 ng/mL IL-1β for 4 h, followed by treatment of the HUVECs with UA/US597 for 24 h. Figure 3C showed that US597 dose-dependently inhibited the mRNA expression of ICAM-1 and E-selectin. In addition, the mRNA expression of VCAM-1 was significantly lower in the US597 (5 μM) treated group than that in the control group (*P* < 0.01). UA (5 μM) inhibited the induction of ICAM-1 and E-selectin mRNA (*P* < 0.05), while the level of VCAM-1 mRNA showed almost no change.

The effects of UA/US597 on the protein expression by HUVECs of ICAM-1, VCAM-1 and E-selectin were evaluated by flow cytometry. As shown in Figure 3A and 3B, US597 at as low as 0.2 μM significantly inhibited ICAM-1 expression (88.05% of control, *P* < 0.05), but only mildly reduced VCAM-1 and E-selectin expression (92.66% and 93.39% of control respectively). High concentration of US597 (5 μM) significantly inhibited ICAM-1, VCAM-1 and E-selectin expression (29.50%, 64.41% and 57.53% of control respectively, *P* < 0.01). However, UA (5 μM) had no significant effect on cell adhesion molecule expression.

To confirm whether UA/US597 could reduce the expression of cell adhesion molecule, we further evaluated the expression of cell adhesion molecule in HUVEC cells by western blot (Figure 3D and 3E). Pretreatment of the HUVECs with US597 at 0.2, 1 and 5 μM significantly inhibited ICAM-1 expression in a dose-dependent manner by 82.59, 53.47 and 25.49% (Figure 3D), respectively, compared to the control. By comparison, E-selectin protein expression was slightly down-regulated in HUVEC cells treated by 5 μM US597 or at 24 h (Figure 3E), but no significant decrease at UA (5 μM) treated group. The result was consistent with that obtained from the flow cytometric analysis. In addition, we also found that 5 μM US597 obviously inhibited the P-selectin protein expression in HUVECs (Figure 3E).

### Analysis of microarray expression data

**Figure 4:**
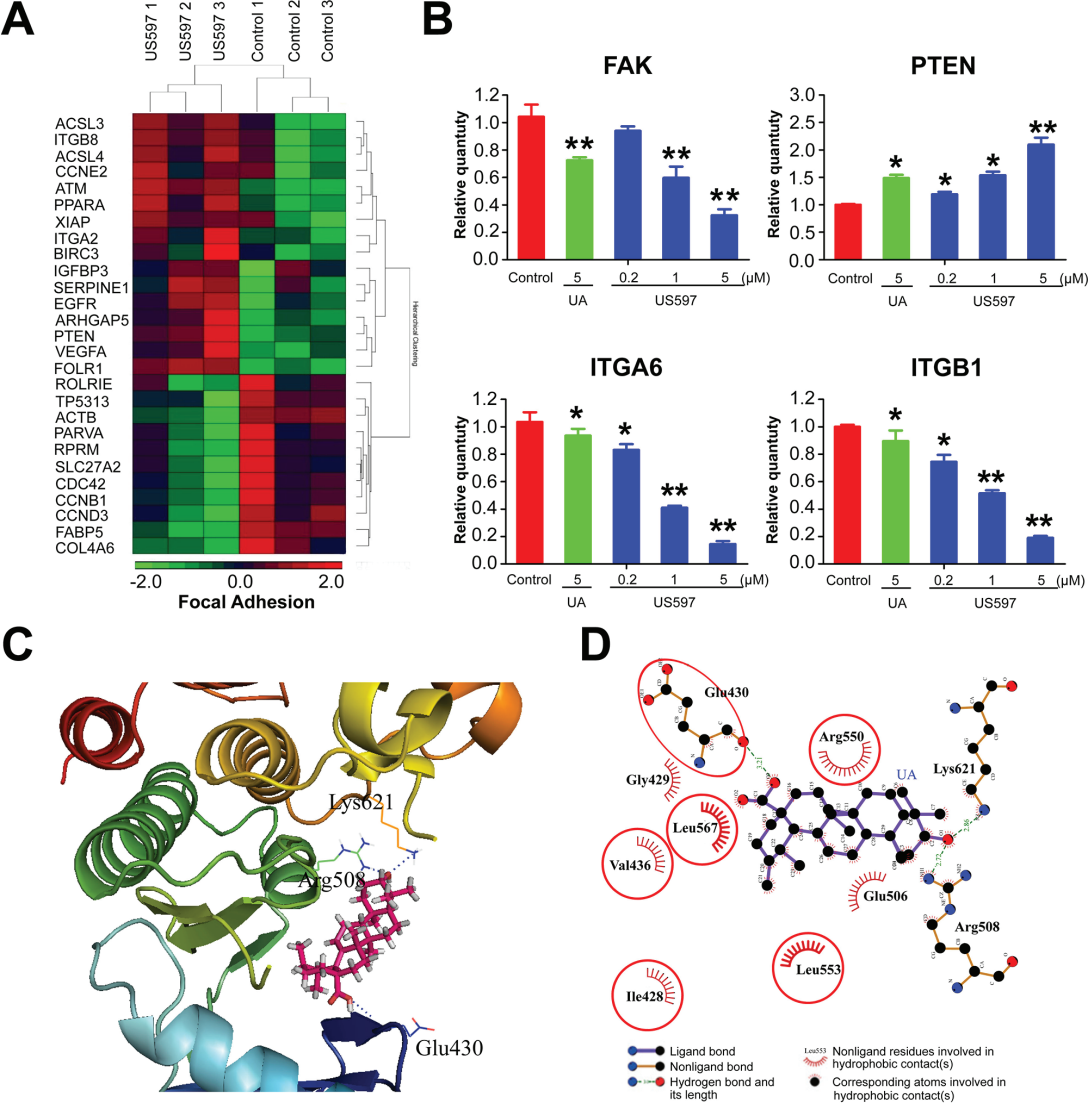
(A) Hierarchical clustering of the gene expression profiles of focal adhesion genes **(B)** Comparison of the expression levels of genes as fold-changes between the control group and the UA/US597 treated group by qRT-PCR. Data were normalized using β-actin as an endogenous control for RNA input. Fold-changes for these mRNAs from the qRT-PCR are shown as mean ± SD. (*n* = 3 for each group, *P < 0.05 and **P < 0.01). **(C)** UA is docked into the active site of FAK, showing interactions between UA and FAK by using the in silico model. **(D)** A 2-dimensional interaction map of UA and FAK.

Considering the facts that UAs have been used in the treatment of liver diseases [24], and hepatocellular carcinoma cell line HepG2 also shows better sensitivity to UA/US597 *in vitro*, we decided to focus on HepG2 cell line for the further underlying mechanism study. To further assess the expression pattern of the genes after HepG2 cells were treated with US597 (5 μM, 24 h), the microarray analysis were performed using an Affymetrix human U133plus 2.0 array. The microarray data showed that the expressions of 720 genes were significantly altered (≥ 1.5 fold change plus corrected *t*-test *p*-value < 0.1): 498 genes were up-regulated and 252 genes were down-regulated (Supplementary Figure S2A, *n* = 3). As indicated in Supplementary Figure S2B, these genes are mostly associated with cell proliferation, apoptosis, adhesion and signal transduction in response to US597 treatment. More importantly, 13 genes were involved in the focal adhesion signaling pathway—the key signaling pathway associated with cancer metastasis (Figure 4A).

To validate the microarray results, we assessed the expression of a subset of focal adhesion genes with qRT-PCR, including: integrin α6 (ITGA 6), integrin β1 (ITGB 1), FAK and PTEN (Figure 4B). The results showed that obvious decreases in integrin α6, integrin β1, FAK and up-regulated PTEN expression were observed in the HepG2 cells treated with US597 in a dose-dependent manner at mRNA levels. Above results confirmed that the microarray data were reliable.

### Molecular docking study on UA/US597

Focal adhesion kinase (FAK), also named Protein-tyrosine kinase 2 (PTK2), is the key regulator of UAs-related proteins involved in focal adhesion pathway, and also the key signal molecule associated with cancer metastasis. To confirm whether UA/US597 could directly inhibit the activity of FAK, molecular docking studies were conducted using the FFT-base protein docking method [25]. We found that UA could dock well into the active site of FAK, which is the key protein involved in cell proliferation, adhesion, migration and invasion. As show in Figure 4C and 4D, the docking data suggested that binding of UA 2 to FAK involved 3 hydrogen bonds Lys621, Arg508 and Glu430, UA also forms hydrophobic interaction with residues Arg550, Ile428, Leu533, Leu567, Glu506, Gly429 and Val436 in the active site of FAK.

### UA/US597 inhibit the integrin α6 and β1 expression in HepG2 cells

**Figure 5:**
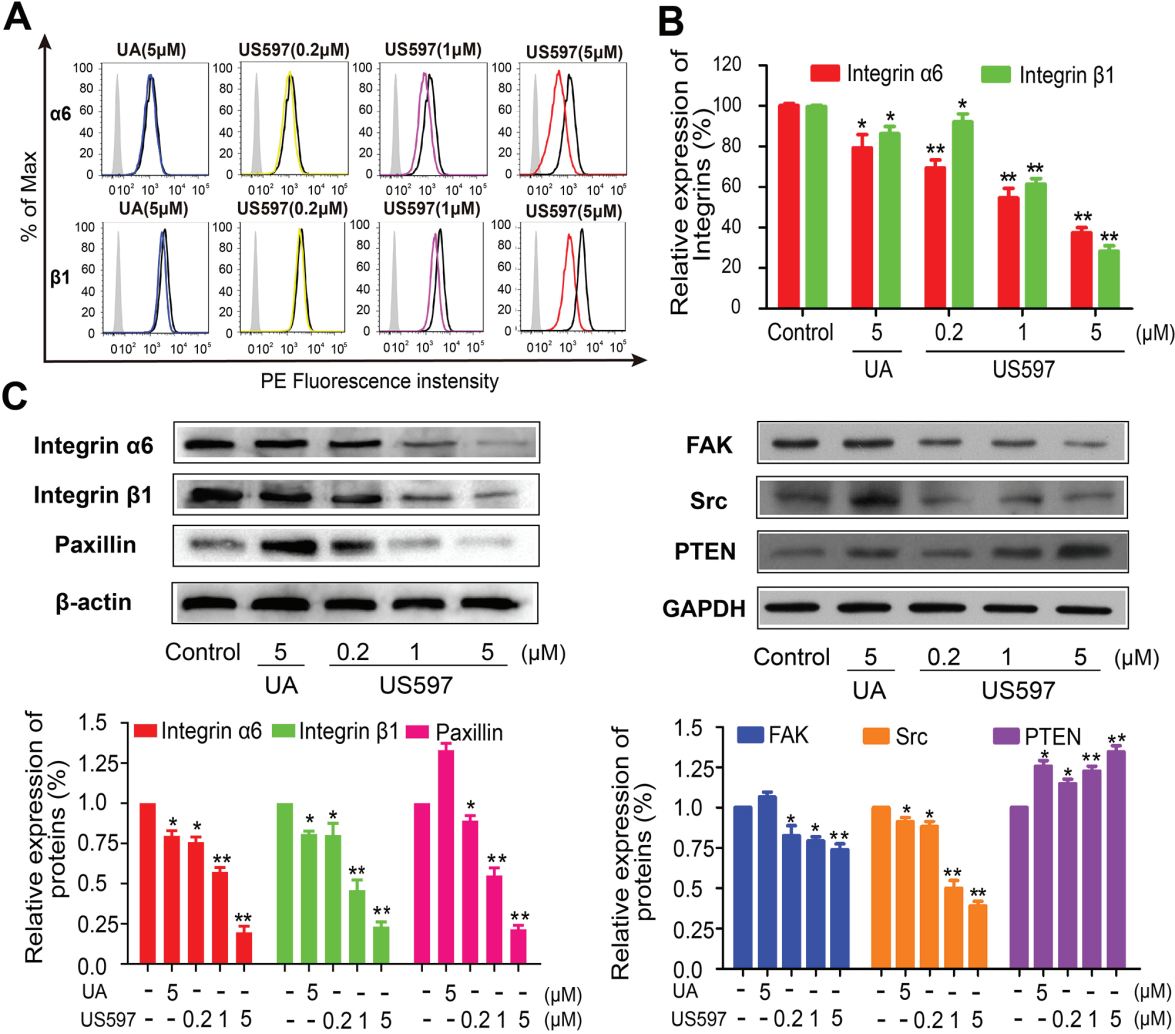
Flow cytometric analysis was performed for integrin α6 and β1 (A) on HepG2 cells, and (B) The inhibitory effect of UA/US597 on the expression of integrin α6 and β1, isotype control (silver area), control (blank curve), blue curve (5 μM UA), yellow, purple and red line represents US597 treated group at concentrations of 0.2, 1 and 5 μM, respectively **(C)** Western blotting analysis of UA/US597 on the down-regulation of integrin α6β1 and its downstream signaling regulator (FAK, Src, paxillin and PTEN) expression in HepG2 cells. Data were obtained from 3 separate experiments and were mean ± SD. (*P < 0.05, **P < 0.01 in comparison with control).

To demonstrate the effect that UA/US597 inhibits the highly expressed adhesion molecules in HepG2 cells, the expressions of α4, α5, α6 and β1 integrin on the surface of HepG2 cells altered by UA/US597 were determined by FACS. Cells were exposed to UA (5 μM) and different concentrations of US597 (0.2, 1 and 5 μM) for 24 h, then used flow cytometry analysis to validate the different levels of protein expression of integrin subunits α6 and β1 (Figure 5A). As shown in Figure 5B, when the HepG2 cells were treated with 0.2, 1 and 5 μM US597, integrin α6 fluorescence intensity were inhibited by 13.95%, 45.33% and 62.52%, respectively, and also caused a significant decrease in integrin β1 expression by 7.92, 38.64, and 71.72%, respectively. In contrast, there were no significant changes in the fluorescence intensity of the α4 (Supplementary Figure S3A) and α5 integrin (Supplementary Figure S3B).

### UA/US597 inhibit integrin-mediated focal adhesion signaling pathway

Because integrin-mediated focal adhesion signaling pathway can influence various processes of tumor cells proliferation, adhesion, invasion and migration, we detected six major groups of proteins including integrin α6, integrin β1, FAK, Src, paxillin and PTEN to detect the effect of UA/US597 on integrin-mediated focal adhesion signaling pathway. As shown in Figure 5C, US597 clearly reduced integrin α6, integrin β1 and downstream FAK, Src and paxillin, up-regulated expression of PTEN in HepG2 in a concentration-dependent manner. When UA was treated, the effect was prominent. Our results showed that UA/US597 can down-regulate the expression of proteins associated with proliferation and metastasis.

### Effect of UA/US597 on experimental lung metastasis and survival

**Figure 6:**
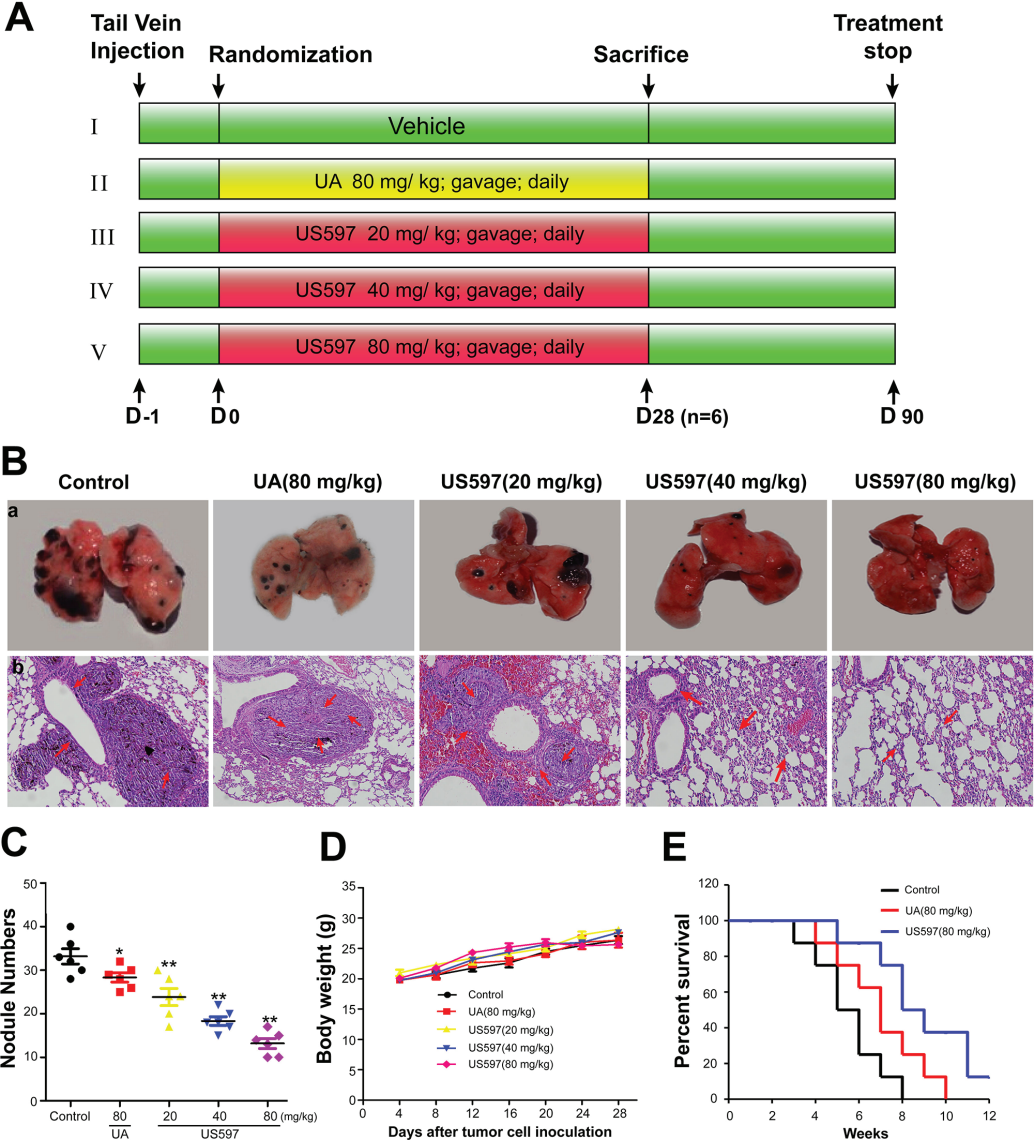
(A) Schematic representation of the experimental protocol described in materials and methods (*n* = 14) **(B)** a, Photography of the lung of animals inoculated with B16-F10 melanoma via tail vein; b, hematoxylin–eosin staining assay represent the metastases in the lungs, the arrows indicate the metastatic area in each group, amplification × 100. **(C)** Metastatic tumor nodules number in lung metastasis model. **(D)** Body weight change during treatment periods. **(E)** Kaplan–Meier survival analysis showed cumulative post-inoculation survival rates of the 80 mg/kg UA/US597 treated mice comparison with the saline treated group. The results were expressed as mean ± SD. *P < 0.05, **P < 0.01 vs control.

After confirming that US597 can significantly inhibited cancer cells adhesion, migration and invasion *in vitro*, we further examined the anti-metastatic efficacy of US597 with a murine artificial pulmonary metastases model *in vivo*. The experimental protocol is depicted in Figure 6A.

Figure 6B (a) showed representative images of pulmonary metastases of B16-F10 melanoma in each treatment group. It was obvious that the mice treated with the US597 showed less pulmonary metastatic nodules than those of mice in control group. Histological examinations showed the presence of melanoma cells in the lung parenchyma, which proved the presence of metastasis (Figure 6B (b)). In addition, the hematoxylin–eosin staining of various lung sections further confirmed that the metastatic nodules colonized in the lungs of US597 treated groups were smaller than that of saline treated group (Figure 6B (b)).

Quantification analysis of the number of nodules was shown in Figure 6C. After 28 days treatment with US597 with 20, 40 or 80 mg/kg/day, the inhibitory rates were 28.31%, 44.58% and 60.24%, respectively. Furthermore, there was no obvious effect on body weights change (Figure 6D) at these dose levels. By comparison, a weaker inhibition (14.45%) was found in UA treatment group. In summary, these results manifested that US597 could efficiently suppress the tumor lung metastasis *in vivo* without obvious side effects in mice.

In addition, the life span of these animals was significantly increased by UA/US597 treatment (80 mg/kg/day). The control animals survived for 8 weeks after inoculation of B16-F10 murine melanoma cells. Animals treated with US597 survived for more than 12 weeks (Figure 6E).

### Histopathological and blood analysis

Our aforementioned results demonstrated that 80 mg/kg UA/US597 by daily oral administration for 28 days, UA/US597 did not induce significant animal body loss. Hence, this dose was chosen to investigate whether UA/US597 can protect against systematic toxicity *in vivo*.

**Figure 7:**
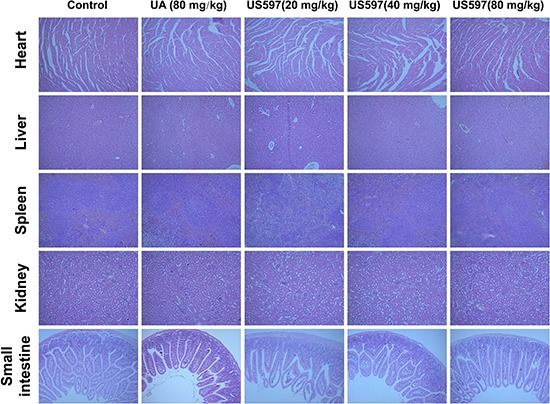
Histological analyses of main viscera tissues of mice Histological section of heart, liver, spleen, kideney and small intestine stained with H&E. Data are representative of at least 3 mice.

After 28 days, normal tissues, such as heart, liver, spleen, kideney and small intestine histopathological photomicrographs were shown in Figure 7. Histological features of control group mouse showed normal structures. Moreover, main viscera tissues including heart, liver, spleen, kidney and small intestine show no remarkable histopathological abnormalities or lesions in UA/US597 treated group.

At the end of the study, the blood toxicity analysis of mice indicated that white blood cell count and lymphocytes decreased significantly in UA/US597 group compared with control group, hemoglobin and platelets were changed in the US597 treated mice compared with the saline treated mice (Table 1). However, all the cell counts remained in the reported normal ranges.

**Table 1:** The blood toxicity of UA/US597-treated tumor metastatic mice

Treatment	RBC (× 10^12^/L)	HGB (× g/L)	WBC (× 10^9^/L)	LYMPH (× 10^9^/L)	PLT (× 10^9^/L)
normal range	6.36 – 9.42	110.0 – 151.0	1.8 – 10.7	0.9 – 9.3	592 – 2972
Control	9.04 ± 0.27	136.42 ± 1.15	9.31 ± 1.28	6.82 ± 2.17	1583 ± 126
UA (80 mg/Kg)	8.84 ± 2.31	131.56 ± 0.89	7.14 ± 0.33^*^	3.65 ± 1.48^**^	1461 ± 251
US597 (80 mg/Kg)	8.56 ± 1.52	117.42 ± 2.17^*^	5.07 ± 2.15^**^	2.86 ± 0.69^**^	1139 ± 164^**^

The blood parameters were analyzed using HEMAVET 950 hematology System.

* indicates significant changes compared to the control group (*P* < 0.05)

** indicates significant changes compared to the control group (*P* < 0.01). RBC, red blood cells; HGB, hemoglobin; WBC, white blood cells; LYMPH, lymphocytes; PLT, platelets.

### The expression of ICAM-1 on the lung tissue of tumor metastatic mice

**Figure 8:**
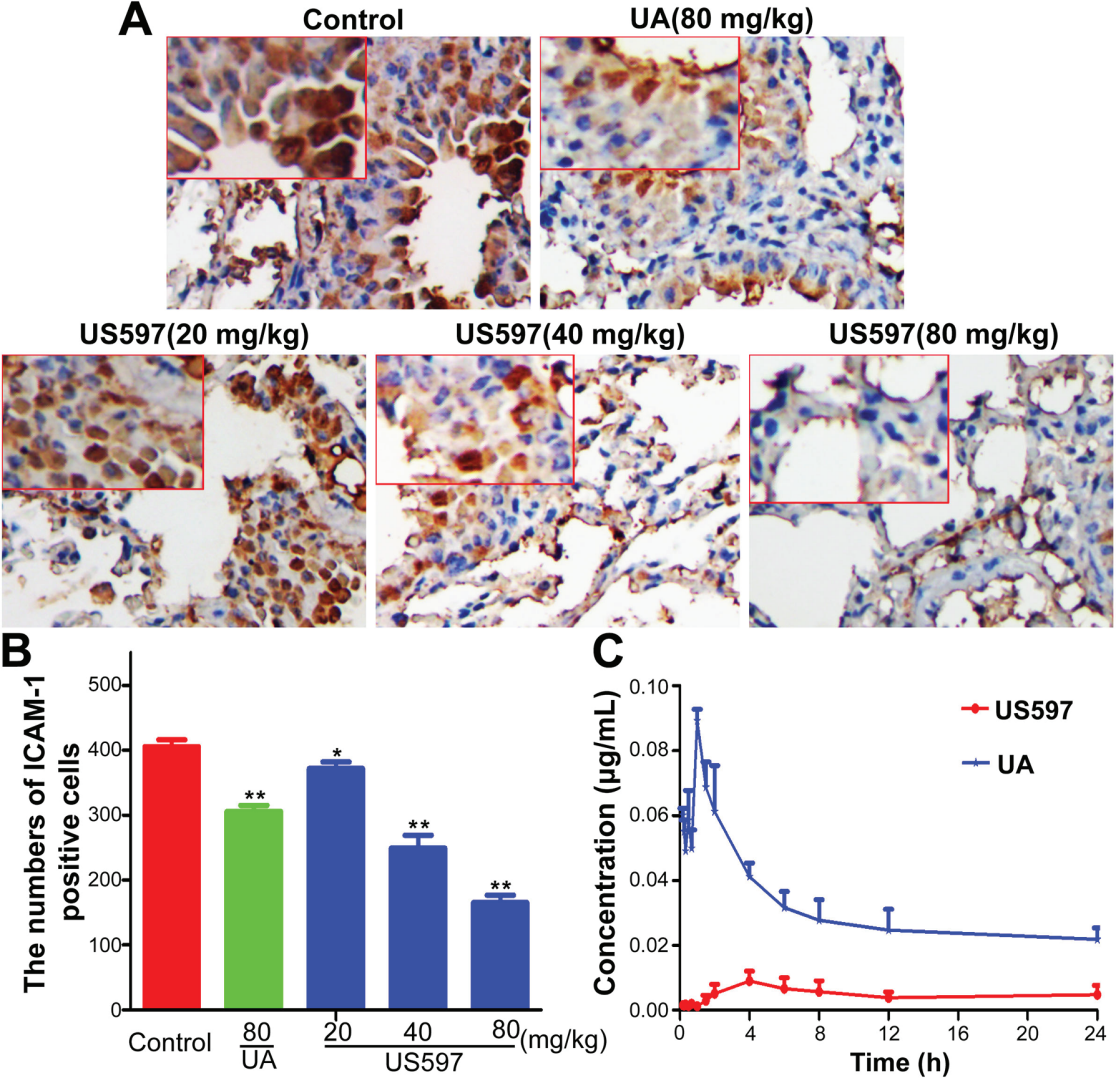
(A) Immunostaining with antibodies to ICAM-1 was performed on lung sections from mice **(B)** Quantitative of the mean ICAM-1 positive area counted at original magnification × 200, the results were expressed as mean ± SD (*n* = 3). *P < 0.05, **P < 0.01 vs control. **(C)** The plasma concentration-time curves of US597 and UA in Sprague-Dawley rats (*n* = 5) after a single oral dose of 80 mg/kg US597.

Inhibition of ICAM-1 expression in lung tissue was determined by immunohistochemical staining (Figure 8A). ICAM-1 expression was appreciably enhanced in endothelial cells of blood vessels in the control group. Compared with the control group, there was no significant changes in the low dose US597 group (*P* > 0.05 Figure 8B). The expressions of ICAM-1 were significantly decreased in the UA and high dose US597 group (*P* < 0.01 Figure 8B).

### Pharmacokinetic characteristics of UA/US597

Our previous study proposed that once endocytosed, US597 may be hydrolyzed by amidase to produce the metabolized product UA *in vivo* [22]. To verify this hypothesis, we performed a pharmacokinetic study to seek if US597 and its parent compound UA are bioavailable in the serum of the mice. After rats received US597 (80 mg/kg) by oral administration, the pharmacokinetic characteristics of US597 and UA were assessed on the same plasma samples, respectively. The mean plasma concentration–time curves of US597 and UA were presented in Figure 8C. As shown in Figure 8C, a significant amount of UA was available in the serum of the mice, and the US597 was observed very well. The key pharmacokinetic parameters of US597 were calculated and summarized in Supplementary Table 1. These results suggested that US597 was mainly distributed in vasculature after oral administration and hydrolyzed by amidase to produce the metabolized product UA *in vivo*, at last eliminated slowly from the body.

## DISCUSSION

In the current work, we reported for the first time the effect of UA and its novel derivative US597 on the hetero-adhesion between human hepatocellular carcinoma cells and endothelial cells. Several studies have documented the anti-cancer activity of UA and its derivatives [20–22]. In the present studies, we showed that US597 had a potential role in inhibiting the growth, adhesion, invasion and migration of SW620, B16-F10 and HepG2 cells *in vitro*, as well as suppressed cancer metastasis *in vivo* by the B16-F10/C57BL/6 mouse melanoma lung metastasis model, suggesting its potential anti-metastasis effect by modulating tumor adhesion and invasion steps both *in vitro* and *in vivo*. Further study showed that the mechanism underlying the above effects of US597 was related, at least in part, to the inhibitory cell-surface CAMs and integrin α6β1 expression in HUVECs and HepG2 cells, respectively.

Metastasis, one of the major causes of morbidity and mortality in cancer patients, is strongly influenced by tumor cell adhesion, migration, and invasion [26]. Adhesion of cancer cells to ECM or vascular endothelium is a crucial starting point of metastasis. Therefore, therapeutic strategies for preventing or suppressing cancer adhesion and metastasis can significantly improve the survival of cancer patients. We found that UA/US597 significantly inhibited cell proliferation in all nine cell lines in a dose-dependent manner (Figure 1 and Supplementary Figure S1). In order to exclude the possible cell cytotoxicity activity, we determined the UA/US597 anti-adhesion, migration and invasion activity at the dose without obvious cytotoxicity (the concentrations lower than its IC50). In the present studies, we demonstrated that UA/US597 could interfere adhesion of HepG2 cells to HUVECs (Figure 2A and 2B) in a concentration dependent manner. Furthermore, tumor migration and invasion, a common feature of tumor cells, are becoming important prerequisites of cancer progression and metastasis [27]. We showed that UA/US597 markedly inhibited the invasive (Figure 2C) and migratory (Figure 2D) abilities of HepG2 cells. In addition, UA/US597 also can inhibit the adhesion, migration and invasion of SW620 and B16-F10 cells at low concentrations. Collectively, the results suggested that UA/US597 could inhibit the adhesion of HepG2 cells to endothelial cells, thus preventing the formation of metastatic foci in the distant organs.

The adhesive interaction between tumor cells and vascular endothelial cells or the extracellular matrix plays a crucial role in metastasis formation. These interactions are mediated by several specific adhesion receptors, such as cellular adhesion molecules, integrins and cadherins [28]. It has become apparent that the expression of cellular adhesion molecules plays a major role in the hetero-adhesion between tumor cells and the vascular endothelial cells over the past few years [3]. Previous studies have shown that various cytokines, such as tumor necrosis factor-α (TNF-α), tumor necrosis factor-α (TNF-β) and interleukin-1β (IL-1β), may stimulate the expression of CAMs (ICAM-1, VCAM-1, E-selectin and P-selectin) by human umbilical vein endothelial cells with different potency [29, 30]. We chose the most potent cytokine IL-1β (1 ng/mL) [29] as a tool molecule in the present study to investigate the effect of UA/US597 on CAMs expression in HUVECs. We found that UA/US597 inhibited the expression of VCAM-1, ICAM-1 and E-selectin in HUVEC cells both at the protein and mRNA levels induced by IL-1β in a dose-dependent manner (Figure 3), indicating that impaired adhesion of HepG2 cells to HUVECs activity may resulted from the downregulation of CAMs expression in HUVECs.

**Figure 9:**
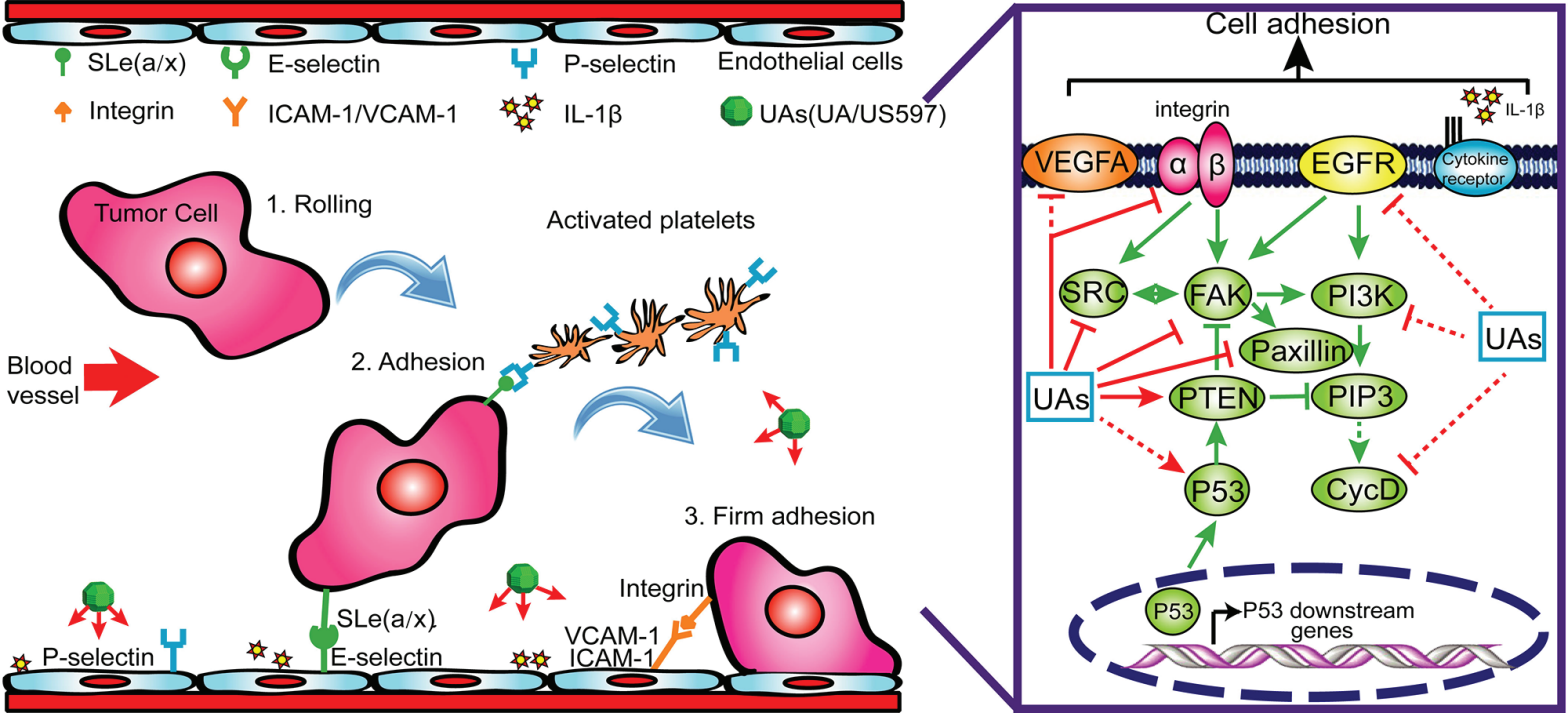
A possible mechanism underlying the inhibitory effect of UA/US597 on cancer metastasis

Tumor invasion and metastasis involve multiple processes, which depend on specific cell-to-cell and cell-to-ECM (extracellular matrix) interactions. These events are mediated in part by integrins that act as receptors for ECM proteins [31]. Integrins are cell-surface glycoprotein receptors that consist of alpha and beta subunits. During cancer metastasis, the expression levels of many different integrins are up-regulated, such as α1β1, α2β1, α3β1, α5β1 and α6β1 [10–12, 32, 33]. Among these, the integrin α6β1, a receptor for laminins, is thought to exclusively bind laminin and to mediate adhesion of human hepatocellular carcinoma cells on substrata coated with this protein [11, 12]. There are reports showing that blocking the expression and activity of integrin α6β1 and its downstream signaling regulator can significantly inhibit hepatocellular carcinoma metastasis [34], which may be related with the inhibition of tumor adhesion, migration, and invasion. In our microarray results, we found 720 genes were altered after US597 treatment (Supplementary Figure S2). Some selected genes were categorized according to their known function. Among them, 13 genes were associated with the focal adhesion, including FAK and PTEN (Figure 4A). We investigated the expression of ITGA6, ITGB1, FAK and PTEN mRNA after UA/US597 treatment of HepG2 cells by qRT-PCR (Figure 4B). In addition, structural docking presents a possible binding mode of UA in the FAK active site (Figure 4C and 4D). In this study, we also examined the inhibition effects of UA/US597 on the expression of integrins (α6β1) and downstream FAK, Src, paxillin and up-regulates PTEN on the surface of HepG2 cells (Figure 5). UA/US597 is able to modulate the expression of proteins linked to proliferation, adhesion, invasion and migration in HepG2 cells (Figure 9). Taken together, our data suggested that the anti-metastatic effect of UA/US597 may be mediated by suppressing the integrin α6β1 and its downstream signaling regulator expression on the cancer cell surface.

A vast majority of previous studies proved that UA and its derivatives could inhibit tumor initiation, growth and progression in a wide variety of preclinical cancer models [17, 35]. Whereas few studies showed that UAs exerted anti-tumor metastasis activity [21, 36]. In our animal study, the anti-metastasis effects of UA/US597 were investigated in a mouse model of established pulmonary metastasis. Tumor metastasis inhibitory effect in mice bearing B16-F10 cells could predict the activity of corresponding tumors in human beings, which closely mimics the stage of metastasis in a lot of human's cancers [37]. The present study clearly showed that the daily administration of UA/US597 resulted in remarkable reduction of lung metastasis (Figure 6B and 6C) and significantly down-regulated ICAM-1 protein expression (Figure 8A and 8B) *in vivo* with a dose-dependent manner. Besides, there is no significant difference between UA/US597 treated and untreated control group in body weights (Figure 6D) and main viscera tissues and blood counts did not exhibit significant abnormalities or lesions (Figure 7 and Table 1). In addition, the Kaplan–Meier estimate showed that the mean survival rates versus time of UA/US597 treated mice were significantly higher than those of the untreated mice (Figure 6E). UA/US597 has the convenience of oral administration, favorable pharmacokinetic properties (Figure 8C and Supplementary Table 1). It was the first time that the UA/US597 was demonstrated to be not only effective in inhibiting melanoma lung metastasis, but also low toxicity to the hosts. UA/US597 may be a safe and effective potential therapeutic agent for patients with early cancer metastasis.

## METHODS

### Materials and reagents

Ursolic acid (UA, purity > 90%) was purchased from Xi'an Ocean Biological Engineering Co., China. Compound US597 was synthesized as previously reported [22]. UA and US597 were dissolved in dimethyl sulfoxide (DMSO) and were used in all experiments. PE-labeled mouse anti-human ICAM-1, VCAM-1, integrin α5, α6 and integrin β1 mAbs, FITC-labeled mouse anti-human integrin α4 mAb, APC-labeled mouse anti-human E-selectin mAb, PE/FITC mouse IgG1 kappa isotype control and APC mouse IgG1 kappa isotype control antibody were all purchased from Becton Dickinson (BD) Pharmingen™. Human interleukin-1 beta (IL-1β) and mouse anti-human beta-actin (β-actin) antibody were purchased from Cell Signaling Technology. Fibronectin, matrigel and endothelial cell growth supplement (ECGS) were obtained from BD Biocoat™. The primary antibodies anti-ICAM-1, anti-integrin α6, anti-integrin β1 anti-E-selectin, anti-FAK, anti-Src, anti-paxillin and anti-PTEN were obtained from Abcam. The secondary antibody goat anti-mouse-IgG horseradish peroxidase was obtained from Promega. All other reagents used in this study were of the highest purity commercially available.

### Cell lines and cell culture

Human hepatic cancer HepG2, MHCC-97H/L cells; colon cancer HT29, SW620 cells; breast cancer MDA-MB-231, MCF-7 cells; melanoma M619, B16-F10 cells and the normal human liver cell line L02 were purchased from the cell Bank of Shanghai Institute of cell Biology. MDA-MB-231 cells were cultured in L-15 medium; MHCC-97H/L, MCF-7 cells were cultured in DMEM medium, and the other cells were cultured in RPMI-1640 medium supplemented with heat-inactivated fetal bovine serum (FBS, 10%), penicillin (100 U/mL), and streptomycin (100 μg/mL) in a humidified atmosphere of 5% CO_2_ at 37°C. Human umbilical vein endothelial cells (HUVECs) were isolated as described elsewhere [38] and cultured on gelatin-coated culture dishes in M199 medium supplemented with 20% heat-inactivated fetal bovine serum, penicillin (100 U/mL), streptomycin (100 μg/mL), and endothelial cell growth supplement (ECGS) (BD Biocoat™) at 37°C and 5% CO_2_. HUVEC between P3 and P5 were used for all experiments.

### Animals

All mouse studies were performed in accordance with animal protocol procedures, approved by the Institutional Animal Care and Use Committee of Fuzhou University. All mice were killed with excess amounts of anesthetic. Female B16-F10/C57BL/6 mice (age 6–8 weeks, body weight 20–25 g) and female Sprague–Dawley rats (body weight 180–220 g) were obtained from the Shanghai Laboratory Animal Center (Shanghai, China), and housed individually in plastic cages at 20°C, with lighting on from 6:00 AM to 6:00 PM. Throughout the experiments, mice were maintained with free access to pellet food and water. All experimental procedures were approved and carried out in compliance with the related ethical regulations of our university. All efforts were made to minimize the animals suffering and to reduce the number of animals used.

### Cell viability measurement

The cytostatic effect of UA/US597 was determined by the MTT assay as previously described [39]. Various cells including MHCC-97H, MHCC-97L, HepG2, L02, M619, B16-F10, SW620, HT29, MCF-7, MDA-MB-231 and HUVECs were treated with indicated concentrations of UA or US597 for 24 hours. Cell viability was determined by detecting the absorbance at 570 nm in a microquant plate reader (Thermo Fisher), triplicate wells were analyzed at each dose.

### Adhesion assay

We used the fluorescence microscope photographed method to evaluate effects of UA/US597 on the anti-adhesion. HUVECs were grown to confluence in 24-well culture plates, pretreated with gelatin for 12 h, and stimulated with IL-1β for 4 h. Rhodamine 123-labeled SW620, B16-F10 and HepG2 cells were co-cultured with the HUVECs monolayers in each well, followed by treatment with UA/US597 for 1 hour. The non-adherent HepG2 cells were removed from the plate by washing with PBS, and the tumor cells bound to the HUVECs were measured by fluorescence microscopy (Zeiss, Germany), we randomly selected 10 visual fields for each well and took pictures under a fluorescence microscope. The total number of 10 visual fields was calculated by using the equation: % adhesion rate of the control = [the number of adhered cells in samples/the number of adhered cells in the control group] × 100%.

### Invasion assay

An invasion assay was carried out using 24-well plate. The 8 μM pore-sized transwell inserts were coated with matrigel at 1 mg/mL on the upper chamber. The lower chamber was filled with medium containing 20% FBS as chemoattractant agents. The coated filter and upper chamber were laid over the lower chamber. SW620, B16−F10 and HepG2 cells were resuspended with reduced serum RPMI 1640 medium and the density was adjusted to 2.5 × 10^5^/mL, and then cell suspension (200 μL) containing different concentrations of UA (5 μM) or US597 (0.2, 1, 5 μM) were seeded onto the upper chamber wells. After incubation for 24 h at 37°C, cells were fixed in methanol for 20 min. The cells on the inner layer were softly removed with a cotton swab, and the adherent cells on undersurface of the insert were stained with 0.1% crystal violet dye for 15 min. The filters were washed with PBS and images were taken. The invading cells were counted and photographed under a light microscope (Zeiss, Germany) at × 200 magnification. Five fields were counted per filter in each group and the experiment was conducted in triplicate.

### Migration assay

SW620, B16-F10 and HepG2 cells (5 × 10^5^ cells per well) were plated in 6-well plates for 24 h, a perpendicular scratch wound was generated by scratching with a 10 μL pipette tip. After rinsing with PBS to remove the detached cells, then incubated with RPMI-1640 medium containing 0.5% FBS and treated with or without UA/US597 for 0 and 24 h. Cells were photographed using a light microscope (Zeiss, Germany) at 0 and 24 h after the drug treatment. The distance that cells migrated through the scribed area was determined by measuring the wound width at 24 h after treatment, and subtracting it from the wound width at 0 h. Experiments were repeated at least three times.

### Flow cytometry

Cells expression of ICAM-1, VCAM-1, E-selectin, integrin α4, α5, α6 and β1 were performed via flow cytometry. Briefly, HUVECs were plated in 6-well plates, grown to 80% confluence, and then pretreated with IL-1β (1 ng/mL) for 4 h, and then incubation with different concentrations of UA/US597 for 24 h. HepG2 cells were grown on 6-well tissue culture plates followed by treatment with different concentration of UA/US597 for 24 h. After incubation, the cells were washed twice with PBS and collected for staining. The cells and primary antibodies were incubated at 4°C for 20 min in the dark. After washing with staining buffer, cell adhesion molecule (ICAM-1, VCAM-1 and E-selectin) and integrins (integrin α6 and β1) expression on cell-surface were measured by BD FACSAriaIII flow cytometry. The data were processed by FlowJo software and expressed as the mean fluorescent intensities.

### Western blotting analysis

HepG2 and HUVECs (pretreated with 1 ng/mL IL-1β for 4 h) cells were treated for 24 h with various concentrations of US597 (0.2, 1 and 5 μM) and 5 μM UA. Cell dishes were washed with cold phosphate-buffered saline and treated with lysis buffer for several seconds at an ice-cold bath, and then cell lysates were clarified by centrifugation at 12,000 g for 5 min under 4°C and the supernatants were recovered. The concentrations of proteins were determined by the BCA method. Samples with equivalent amounts of proteins were resolved by SDS-PAGE using a 10% gel. Proteins were transferred to a polyvinyldene difluoride (PVDF) membrane. The membranes were washed for ten minutes with TBS (20 mM Tris, 160 mM NaCl) and were blocked by blocking solution (10 mM Tris-HCl (pH 7.4), 125 mM NaCl, 0.1% Tween 20, and 5% skim milk). The blots were washed three times with TBST(20 mM Tris, 160 mM NaCl, 0.1% Tween 20), and they were incubated with specific primary antibodies diluted 1:1000 in TBST solution overnight at 4°C. Subsequently, the blots were washed three times with TBST (20 mM Tris, 160 mM NaCl, 0.1% Tween 20), and then the blots were incubated with secondary antibody (horseradish peroxidase conjugated goat anti-rabbit-IgG) diluted 1:5000 in TBST solution for 2 h at 37°C. The membranes were washed with TBST and TBS. Chemiluminescent signals were generated using a Super Signal West Pico Chemiluminescent Substrate kit (Pierce), and detected by using the ChemiDoc XRS system (Bio-Rad). The target proteins expression was quantified by use of Image Lab analysis software (Bio-Rad).

### Molecular docking study

To understand the molecular interaction between UA/US597 and focal adhesion kinase (FAK), the docking method was similar to that described previously [22]. The crystal structure of FAK (PTK2) was used as the docking proteins [40]. The docking experiment was carried out by using DFT/6–31G in Gaussian 09 [41]. All parameters were calculated with the default protocols unless otherwise noted.

### Microarray assay of gene expression

HepG2 cells were cultured in medium containing 5 μM US597 for 24 h, and cells incubated in medium without US597 acted as a control. Total RNA was extracted from HepG2 cells (2 × 10^6^) using TRIzol reagent (Invitrogen) and mRNA microarray analyses using Affymetrix GeneChip U133plus2.0 Array (Affymetrix, Santa Clara, CA) were performed according to manufacturer's instructions. Microarrays were scanned by using Affymetrix^®^ GeneChip Command Console (AGCC) which installed in GeneChip^®^ Scanner 3000. The data were analyzed with Robust Multichip Analysis (RMA) algorithm using default analysis settings and global scaling as normalization method by Partek^®^ Genomics Suite 6.6. Pathway analysis of the genes obtained from the Natural Language Processing (NLP) analysis. Values presented are log2 RMA signal intensity.

### Total RNA isolation and quantitative real-time PCR

Total RNA was isolated from cells in control group and UA/US597 treated groups at different concentrations, using Trizol reagent (Invitrogen) according to the manufacturer's protocol. A sample (1 μg) of total RNA was used for the synthesis of the first strand cDNA using the PrimeScript^®^ RT reagent Kit (Takara, Dalian, China) according to the manufacturer's instructions. The PCR was carried out with 1μL cDNA in a 25 μL reaction volume using a SYBR^®^ Premix Ex Taq™ PCR kit (Takara, Dalian, China). The sequences are 5′-GATTGTCATCACCACTGTGGT AGC-3′ and 5′-GGCCTGTTGTAGTCTGTATTT CTT-3′ for ICAM-1, 5′-GACCACATCTACGCTGAC-3′ and 5′-GCAACTGAACACTTGACTG-3′ for VCAM-1, 5′-GGATGATGCCTACTTGTGAAG-3′ and 5′-CAGGAGCCAGAGGAGAAATG-3′ for E-selectin, 5′-GTGCTCTTGGTTCAAGCTGGAT-3′ and 5′-ACTTGAGTGAAGTCAGCAAGATGTGT-3′ for FAK, 5′-GGACGAACTGGTGTAATGATATG-3′ and 5′-TCTACT GTTTTTGTGAAGTACAGC-3′ for PETN, 5′-TCCCTGAACCTAACGGAGTCT-3′ and 5′-ATGTCCAAGTAGTTCAGTTTG-3′ for integrin α6 (ITGA6), 5′-ACAGCAGAG AAGCTGAAGCCA-3′ and 5′-GAGCTTAGCTGGTGTTGTGC-3′ for integrin β1 (ITGB1), 5′-TGGCACCCAGCACAATGAA-3′ and 5′-CATAGTCATAGTCCGCCTAGAAGCA-3′ for β-actin. Quantitative real-time PCR (qRT-PCR) was performed by using SYBR-Green real-time PCR methodon the CFX96^™^ Real-Time PCR Detection Systems (Bio-Rad). Target mRNA expression levels were normalized with the β-actin mRNA and calculated by the 2-ΔΔCt method. Results are presented as the mean ± standard deviation of three independent experiments.

### *In vivo* tumor growth and metastasis assay

To establish the lung metastasis model, B16-F10 cells were harvested, washed with serum-free RPMI 1640 and resuspended to give the appropriate concentrations in PBS, and injected into the tail vein of C57BL/6 mice at a density of 5 × 10^4^/200 μL. On the following day (Day 1), mice were randomly divided into five groups (*n* = 14 for each group), and then they were orally gavaged with either normal saline (control), UA (80 mg/kg) and US597 (20, 40 and 80 mg/kg) for 28 days, respectively. Mice weight was measured every four days for 7 times. Mice were sacrificed (*n* = 6) at the 28st days after tumor inoculation. The heart, liver, spleen, lung, kidney and small intestine were excised, washed with PBS, and fixed in 10% neutral buffered formalin, and these tissues were then paraffin embeddedand stained with hematoxylin and eosin (H&E). The number of surface tumor nodules was determined by visual inspection using a magnifying glass, and histological observations were performed under a microscope (Zeiss, Germany). The other eight animals were observed for more than 12 weeks (D 90) for a determination of survival rate in control and UA/US597 treated groups, respectively.

### ICAM-1 immunohistochemistry

Frozen sections (6 μm) were stained with anti-ICAM-1 antibody as previously described [42]. ICAM-1 expression in lung tissue was performed with avidin-biotin complex (ABC) method. Results for ICAM-1 positive cells are shown as × 200 magnification.

### Pharmacokinetic assessments

The pharmacokinetic properties of US597 were investigated in male Sprague–Dawley rats (Shanghai Laboratory Animal Center). On the study day, the rats received an 80 mg/kg dose of US597 by oral administration. The pharmacokinetic study involved serial arterial blood sampling (500 μL) with 13 samples obtained from each animal at 0.17, 0.25, 0.33, 0.5, 0.67, 1, 1.5, 2, 4, 6, 8, 12 and 24 h after administration. The plasma concentrations were determined by UPLC-MS/MS analysis (Waters Corp., Milford, MA, USA). Noncompartmental pharmacokinetic parameters were fitted using DAS software (Data Analysis System 3.0, BioGuider Co., Shanghai of China).

### Statistical analysis

Data represents the means ± standard deviations (SD) for three independent experiments. Statistical analysis was performed using the Student's *t*-test and one-way analysis of variance. Multiple comparisons between the means were done by the least significance difference (LSD) test. A probability (*p*) value < 0.05 was considered statistically significant, and *P* < 0.01 was highly statistical significance. All statistical analyses were performed employing the SPSS statistical software (version 19.0).

### Author's contributions

JWS and LPX designed research, wrote and revised the manuscript. JWS and LPX developed methodology, collected, analyzed and interpreted data; LPX, XFC, JZC, TC and QT performed the experiments. XY and XBY acquired figures. LJ and RJH reviewed the paper.

## CONCLUSIONS

In summary, the present study for the first time demonstrated that UA and its novel derivative US597 were able to suppress cell surface adhesive molecules including ICAM-1, VCAM-1, E-selectin and P-selectin proteins expression, and further target integrin α6β1-mediated focal adhesion signaling pathway (Figure 9), and resulted in inhibiting adhesion and migration of cancer cells *in vitro* and *in vivo*. More importantly, UA/US597 has low toxicity to the hosts. These findings confirmed that UA and US597 were novel promising anti-metastasis drugs, particularly as new potential chemopreventive agents for suppressing hepatocellular carcinoma recurrence and early metastasis.

## SUPPLEMENTARY FIGURES AND TABLE


