# Supplementary material for: A pentacyclic triterpene natural product, ursolic acid and its prodrug US597 inhibit targets within cell adhesion pathway and prevent cancer metastasis
Source: Oncotarget. 2015 Mar 19;6(11):9295–312. doi: 10.18632/oncotarget.3261 (PMC4496218; doi:10.18632/oncotarget.3261)
Supplement: Supplementary file 1 [file oncotarget-06-9295-s001.pdf]

SUPPLEMENTARY FIGURES AND TABLE

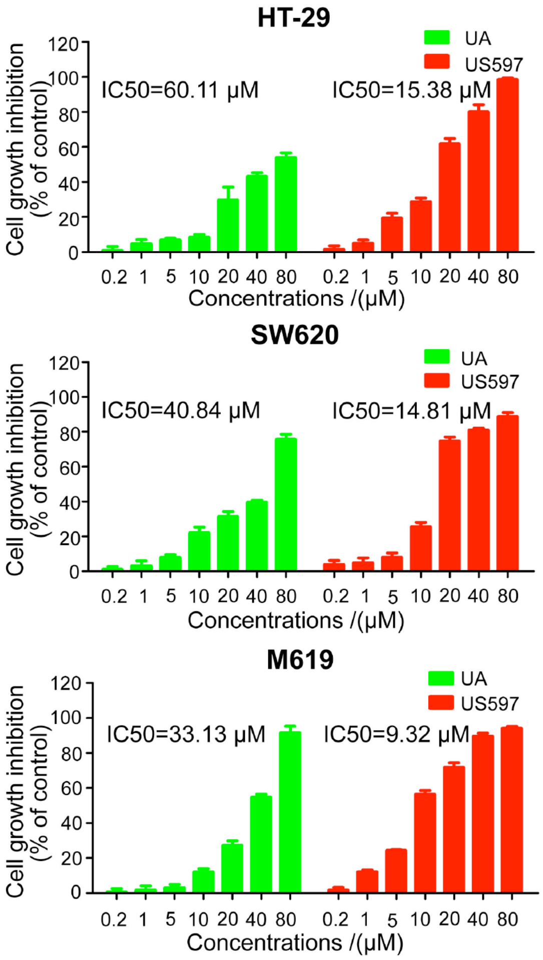

Supplementary Figure S1: HT29, SW620, MDA-MB-232, MCF-7 and M619 cells were treated with the indicated concentrations of UA/US597 for 24 h, and cell viability was evaluated by the MTT assay. Results are shown as mean ± SD from three separate experiments in each experiment.

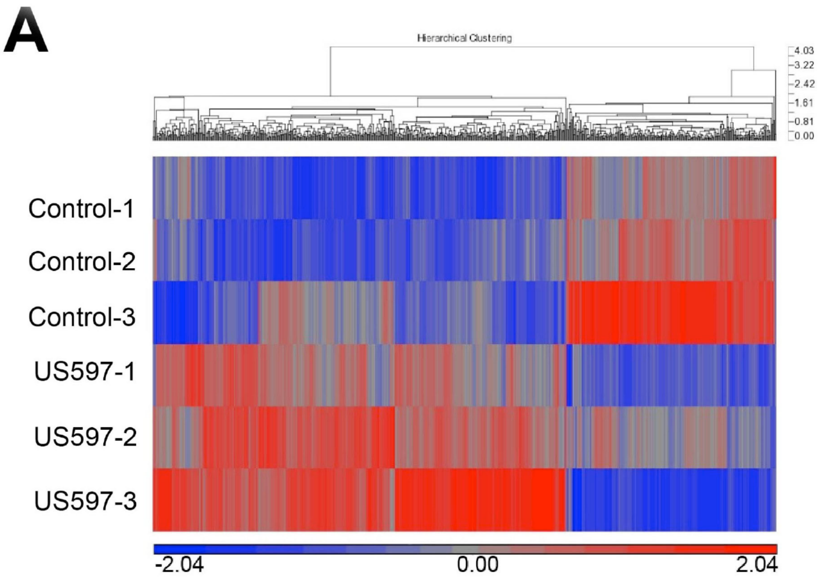

B

Pathway description	Count	P-Value	Genes name
p53 signaling pathway	9	2.45E-03	CCNE2, CCNB1, TP53I3, CCND3, SERPINE1, RPRM, IGFBP3, PTEN, ATM
Lysosome	10	2.14E-02	LAMP2, SLC17A5, TPP1, AP3M1, HEXB, NEU1, CTSA, CD164, ASAH1, CTSL1
Focal adhesion	13	4.99E-02	ACTB, EGFR, XIAP, ITGA2, BIRC3, PTEN, COL4A6, CDC42, ARHGAP5, CCND3, ITGB8, VEGFA, PARVA
Amino sugar and nucleotide sugar metabolism	5	6.76E-02	PGM3, GNPDA1, MPI, GFPT1, HEXB
Valine, leucine and isoleucine degradation	5	6.76E-02	MCCC2, IVD, ALDH1B1, HMGCS1, PCCB
Small cell lung cancer	7	7.44E-02	CCNE2, XIAP, SKP2, ITGA2, BIRC3, PTEN, COL4A6
Hypertrophic cardiomyopathy (HCM)	7	7.78E-02	ACTB, ACTC1, ITGB8, ITGA2, PRKAA2, TPM1, TPM3
Alanine, aspartate and glutamate metabolism	4	9.39E-02	GLUL, ALDH5A1, GFPT1, ASNS
PPAR signaling pathway	6	9.45E-02	PPARA, OLR1, ACSL4, ACSL3, SLC27A2, FABP5
Terpenoid backbone biosynthesis	3	9.59E-02	HMGCR, HMGCS1, IDI1

Supplementary Figure S2: (A) Heat map depicting microarray gene expression profiling in HepG2 cells treated with 5 µM US597 and compared with control. (B) Pathway analysis of the genes obtained from the NLP analysis. There were 134 pathways revealed. Among them, about 11 signaling pathways were significant ($P < 0.01$).

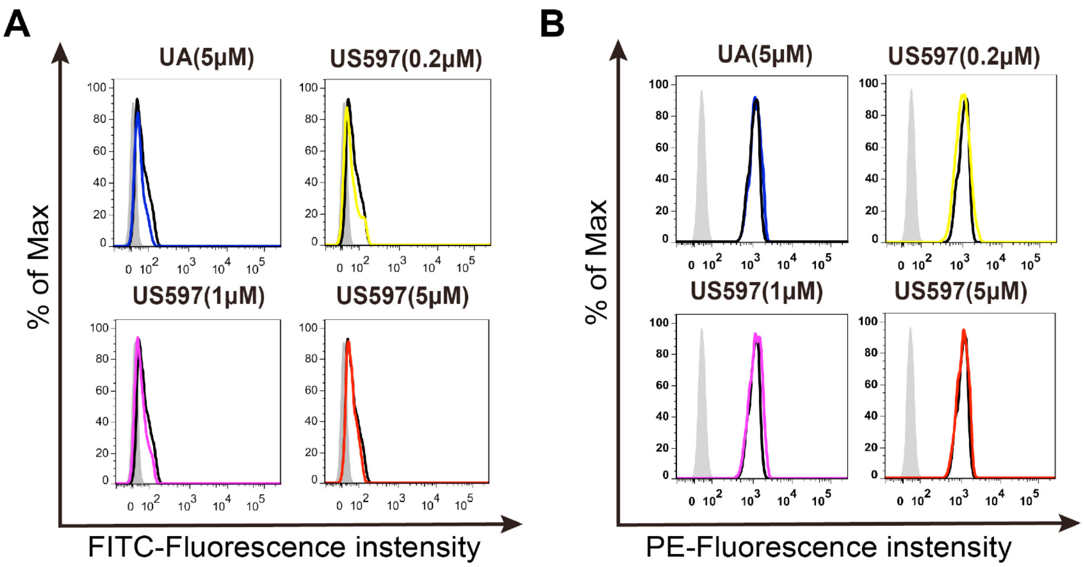

Supplementary Figure S3: Flow cytometric analysis the inhibitory effect of UA/US597 on the expression of integrin α4 (A) and α5 (B) on HepG2 cells, isotype control (silver area), control (blank curve), blue curve (5 μM UA), yellow, purple and red line represents US597 treated group at concentrations of 0.2, 1 and 5 μM, respectively.

Supplementary Table S1: Pharmacokinetic parameters of US597 after oral administration to SD rats (n = 5)

Parameters	mean ± SD
C_{max} (μg/mL)	0.014 ± 0.011
T_{max} (h)	5.6 ± 3.578
$t_{1/2}$ (h)	13.173 ± 9.533
$AUC_{(0–t)}$ (μg·h/mL)	0.155 ± 0.108
$AUC_{(0–\infty)}$ (μg·h/mL)	0.195 ± 0.099
CL(mL/h/mg)	501.275 ± 249.729
V_d (mL/g)	9097.777 ± 6149.829
$MRT_{0–t}$ (h)	11.109 ± 1.776
$MRT_{0–\infty}$ (h)	20.56 ± 10.797
